# Supplementary material for: Social anxiety in young people: A prevalence study in seven countries
Source: PLoS One. 2020 Sep 17;15(9):e0239133. doi: 10.1371/journal.pone.0239133 (PMC7498107; doi:10.1371/journal.pone.0239133)
Supplement: Attachment — Submitted filename: Response to Reviewers file.docx [file pone.0239133.s001.docx]

Dear Prof. Lincoln and the reviewers,

Thank you for reading our manuscript and providing the thoughtful comments. We have addressed each concern, describing each change below and where it occurs in the manuscript. We have now also processed the figures using the online PACE tool. Thank you for your reflections and suggestions.

Sincerely,

P. Jefferies and M. Ungar

Additional Editor Comments:

Thank you for your submission. The reviewers felt like this paper is important for the field, but they have some reservations about various aspects of the manuscript. We invite you to revise and resubmit.

Journal requirements: When submitting your revision, we need you to address these additional requirements.

1. Please ensure that your manuscript meets PLOS ONE's style requirements, including those for file naming. The PLOS ONE style templates can be found at

https://journals.plos.org/plosone/s/file?id=wjVg/PLOSOne_formatting_sample_main_body.pdf and

https://journals.plos.org/plosone/s/file?id=ba62/PLOSOne_formatting_sample_title_authors_affiliations.pdf

Thank you for guiding us to these templates. We have revised the figures and tables and updated the Acknowledgements section to suit journal requirements.

2. We note that you have stated that you will provide repository information for your data at acceptance. Should your manuscript be accepted for publication, we will hold it until you provide the relevant accession numbers or DOIs necessary to access your data. If you wish to make changes to your Data Availability statement, please describe these changes in your cover letter and we will update your Data Availability statement to reflect the information you provide.

We have re-uploaded a copy of the data to the OSF, which can be accessed using the following link: <https://osf.io/vcnf7/?view_only=990d3a095ffa44ffbc40a370be6783c8>. The general project link is: <https://osf.io/vcnf7/> and the DOI is **10.17605/OSF.IO/VCNF7**. We would kindly request that these links and the data are not shared further until/pending acceptance.

3. Thank you for stating the following in the Acknowledgments Section of your manuscript:

'The authors would like to acknowledge the role of Edelman Intelligence for collecting the data and Unilever and CLEAR for funding and commissioning the overarching project as part of their mission to support the resilience of young people experiencing social anxiety.'

We note that you have provided funding information that is not currently declared in your Funding Statement. However, funding information should not appear in the Acknowledgments section or other areas of your manuscript. We will only publish funding information present in the Funding Statement section of the online submission form.

Please remove any funding-related text from the manuscript and let us know how you would like to update your Funding Statement. Currently, your Funding Statement reads as follows:

'The author(s) received no specific funding for this work.'

Additionally, because some of your funding information pertains to commercial funding, we ask you to provide an updated Competing Interests statement, declaring all sources of commercial funding.

In your Competing Interests statement, please confirm that your commercial funding does not alter your adherence to PLOS ONE Editorial policies and criteria by including the following statement: "This does not alter our adherence to PLOS ONE policies on sharing data and materials.” as detailed online in our guide for authors  [http://journals.plos.org/plosone/s/competing-interests](about:blank).  If this statement is not true and your adherence to PLOS policies on sharing data and materials is altered, please explain how.

Please include the updated Competing Interests Statement and Funding Statement in your cover letter. We will change the online submission form on your behalf.

 Please know it is PLOS ONE policy for corresponding authors to declare, on behalf of all authors, all potential competing interests for the purposes of transparency. PLOS defines a competing interest as anything that interferes with, or could reasonably be perceived as interfering with, the full and objective presentation, peer review, editorial decision-making, or publication of research or non-research articles submitted to one of the journals. Competing interests can be financial or non-financial, professional, or personal. Competing interests can arise in relationship to an organization or another person. Please follow this link to our website for more details on competing interests: [http://journals.plos.org/plosone/s/competing-interests](about:blank)

We have now removed the statement in the Acknowledgement section regarding funding information, per this point and the guidance in the first link of comment 1.

The Funding Statement should be fine to leave as is, as we received no specific funding for this study. The previous note about funding mistakenly referred to the original data collection, which was performed independently.

For the Competing Interests, could we just clarify what is required based on our situation? We reviewed the journal’s policy and definition and are not sure whether a CI statement applies, but of course are happy to complete one if it does. In this study, we are performing secondary analyses of a previously collected dataset. We received no specific funding to do these secondary analyses. The original project to collect the data was performed independently. However, the lead author is on a Fellowship scheme which is jointly funded by an academic institution and an industry partner, the latter of which is associated with the organisation who collected the data in the first place. Both entities (academic institution and industry partner) provide the funding for the fellowship, but do not direct or influence any studies undertaken by the researcher.

4. Please amend your manuscript to include your abstract after the title page.

This is now included.

[Note: HTML markup is below. Please do not edit.]

Reviewers' comments:

Reviewer's Responses to Questions

**Comments to the Author**

1. Is the manuscript technically sound, and do the data support the conclusions?

The manuscript must describe a technically sound piece of scientific research with data that supports the conclusions. Experiments must have been conducted rigorously, with appropriate controls, replication, and sample sizes. The conclusions must be drawn appropriately based on the data presented.

Reviewer #1: Yes

Reviewer #2: Yes

2. Has the statistical analysis been performed appropriately and rigorously?

Reviewer #1: Yes

Reviewer #2: Yes

3. Have the authors made all data underlying the findings in their manuscript fully available?

The [PLOS Data policy](http://www.plosone.org/static/policies.action#sharing) requires authors to make all data underlying the findings described in their manuscript fully available without restriction, with rare exception (please refer to the Data Availability Statement in the manuscript PDF file). The data should be provided as part of the manuscript or its supporting information, or deposited to a public repository. For example, in addition to summary statistics, the data points behind means, medians and variance measures should be available. If there are restrictions on publicly sharing data—e.g. participant privacy or use of data from a third party—those must be specified.

Reviewer #1: Yes

Reviewer #2: Yes

4. Is the manuscript presented in an intelligible fashion and written in standard English?

PLOS ONE does not copyedit accepted manuscripts, so the language in submitted articles must be clear, correct, and unambiguous. Any typographical or grammatical errors should be corrected at revision, so please note any specific errors here.

Reviewer #1: Yes

Reviewer #2: Yes

5. Review Comments to the Author

Please use the space provided to explain your answers to the questions above. You may also include additional comments for the author, including concerns about dual publication, research ethics, or publication ethics. (Please upload your review as an attachment if it exceeds 20,000 characters)

Reviewer #1: This study provides valuable information for both researchers and clinicians specializing in anxiety as it offers an examination of cross-cultural and worldly prevalence rates of SAD. In addition to examining prevalence rates between seven countries, the authors examine SAD rates through contributing factors such as gender, age, and SES. The strengths of this paper include the evolutionary perspective of social anxiety and making the distinction between subclinical social anxiety and symptoms that reach the clinical threshold of SAD. The authors also present multiple graphs of their findings that are visually appealing and easy to understand. There are a number of suggested changes that would strengthen this manuscript, which are presented below.

Thank you for taking the time to review our manuscript, and for the constructive comments. We have addressed each of the comments below, making significant changes to the content of the manuscript.

Major:

1. The authors can improve the quality of this manuscript by expanding on many ideas and theories that are only briefly mentioned. This can be accomplished both by incorporating more background literature as well as the authors’ own interpretations and hypotheses. These include:

Introduction:

-Speak more to the distinction between subthreshold social anxiety and clinical levels of SAD. For example, are there any predisposing factors for some to reach clinical levels of social anxiety compared to others? Are there any current theories (potentially physiological in nature) that explain the differences between subclinical and clinical social anxiety?

We have now included a section in the introduction that clarifies that more studies are now considering social anxiety in terms of a spectrum, to take into account those experiencing more moderate levels of social anxiety (subclinical/subthreshold) where impairment is also pronounced and wide-ranging (like SAD). Our understanding is that this literature is emerging, and robust theories differentiating why some experience subclinical/subthreshold levels of social anxiety and others experience SAD have not yet been articulated or widely accepted. However, we also have a new section later in the introduction, due to your later comment, which describes developmental theories of SAD that suggest development-related factors predispose an individual to vulnerability (see later), which may be more pronounced in some than others, and which may therefore lead to higher or lower levels of social anxiety.

-When discussing current prevalence rates of SAD, the authors should consider also including rates of undiagnosed SAD and implications on treatment. The authors first include this information in the discussion starting on line 331 but it should be first presented in the introduction.

Thank you for the suggestion. We have included a line in the introduction noting that SAD is often undiagnosed, which may contribute to underreporting of prevalence rates.

-It may be helpful to include a developmental theory of SAD in the introduction, especially as the authors repeatedly reference social media and technology contributing to the development of SAD in young adults.

Thank you for this suggestion. We agree and following mention of the prevalence rates of social anxiety, where it is described that young people are disproportionately affected, we have now included a potential explanation based on the work of Leigh and Clark (cited within the paper), indicating social and neurocognitive factors developing at this time that predispose individuals to social anxiety, and that some are at greater risk during this period.

-Likewise, given the focus on worldly prevalence of SAD, further detail on cross-cultural perspectives is likely merited. The authors briefly social anxiety in Asian cultures but should offer more information regarding other cultures. Additionally, the authors could provide hypotheses from the existing literature as to why there are differing rates of social anxiety in different parts of the world rather than simply stating the statistics in the introduction (i.e., the differences in social anxiety expression that the authors note in the discussion). For example, the authors find that speaking with authority figures to be a top concern for Asian countries compared to other countries, which may potentially be linked with cultural values of respect to authority and status that could discussed in the introduction.

We agree that cross-cultural differences merit further discussion. To keep the focus on young people and social anxiety, rather than include this in the introduction, we have included a greater exploration of potential reasons for country differences in the discussion (see later).

-It would be helpful for readers to have some explanation (either hypothesized by the existing literature or the authors’ own speculation) as to why social anxiety rates are increased by social media/technology and why this rate is hypothesized to increase for only young adults. This may be achieved through the Lecrubier et al. and Leigh and Clark’s theories that are referenced in the discussion.

Thank you for this suggestion. We have included further content in the introduction to expand on the relationship between social media/technology and social anxiety. We have included some new studies that suggest potential mechanisms and then speculated as to why this could be particularly challenging for young people. In particular, it builds on the Leigh and Clark theory mentioned earlier about the pivotal nature of social relationships in adolescence and how social media/technology may interfere with this critical development period.

-The authors introduce contributing factors of status, location, and education in the findings but do not discuss these factors in the introduction (i.e., line 315-316 females scoring higher on SAD measures than males). Consider presenting these factors and their associated literature earlier on in the manuscript given they are major areas of the study’s focus.

Thank you for foregrounding the importance of this. We first mentioned these factors in the method section of the paper, but have now also included them in the introduction, at the end of the paragraph which describes prevalence rates.

-The authors do not provide a reason or explanation for their study. Consider clarifying the importance of this particular study and reasoning behind specific aims (Why young adults with SAD? Why assess cross-cultural prevalence?). Was there any reasoning behind choosing the seven countries that were included in this study?

We agree that the importance of the study needs greater foregrounding. We have refreshed and combined the two final paragraphs of the introduction to make clear the need for the study, which is foremostly to determine whether social anxiety is more widespread in young people than previously reported, given suggestions that it may be increasing. The seven countries were chosen by the organisation who conducted the original data gathering project (ours was a secondary analysis of their data) because they are target markets for their brands. However, they are interesting comparators given not all are in the West or Asia, all in the Northern/Southern hemisphere, etc.

Discussion:

-Before line 299, it is suggested that the authors first reiterate and elaborate on their findings regarding age and social anxiety before their discussion of these particular findings. The authors may want to further elaborate on their findings of age and social anxiety. Do these findings predict an increase in the rates of SAD in the future? What are the clinical implications of this potential high-risk age range?

In the discussion we have now noted that 18-24 year olds had significantly higher levels of social anxiety than 16-17 and 25-29 year olds, which supports the developmental theories regarding greater dependence on social relations beyond the family unit.

We have also clarified in the conclusion that it is this 18-24 year old group that are most at risk. Given our study does not include measures of social anxiety, we have avoided speculating on a further increase in the prevalence of social anxiety in the future. Instead, we provide an update to current social anxiety prevalence figures which we hope leads to greater awareness and further investigation.

-Expand on findings related to cross-cultural differences in prevalence (paragraph starting line 307). Why might these discrepancies exist? What are the implications of this finding? Additionally, the two sentences starting at line 309 (Our findings concur…) ending at line 313 are confusing to the reader. Consider rephrasing or possibly listing countries with the highest to lowest rates of endorsement for social anxiety.

Thank you for pointing out the lack of clarity in this section. We have now rephrased it so it makes clear that our findings are in agreement with those of Hofmann and colleagues in terms of high rates in the US, but then when Hofmann and colleagues suggest Russia has a similarly high prevalence and that Asian countries are lower, that our findings differ. We also refer readers to the Table displaying these country differences so they can see how each compares.

We have also extended this section to address cross-cultural differences. We have avoided speculating reasons for differences between cultures, instead drawing focus to our findings about the types of concerns (different social situations) which the SIAS probes, which may account for differences (e.g., Asian countries rating speaking with individuals in authority as a significant challenge, while other countries do not). In this way, differences may potentially be explained by differences in responses to specific social situations, which we have noted is an interesting opportunity for a further study to confirm.

-Explain potential reasoning behind the differences seen in male/female prevalence rates (paragraph starting line 314). Are these findings in line with the existing literature on gender differences in prevalence rates in these respective countries? Why might these differences exist in this particular sample?

The literature generally suggests that females have higher social anxiety than males, but our study shows no difference at the overall dataset level, and then when breaking down by country, two show a difference, four do not, and one shows the opposite (males having higher social anxiety than females). This suggests that a sex difference is probably nuanced across countries and we have refreshed this section to clarify this. Rather than engaging too much speculation about culture and sex, we have tentatively suggested that explanations for sex-related social anxiety differences appear to relate to gender, and therefore the potential way that gender is constructed in different countries may account for differences, and also if gender roles in some countries are becoming less fixed or extreme, that this may also account for the discrepancy between previous findings and this study, especially as young people are likely to reflect societal changes like this first.

-The authors should consider including additional implications of incorrectly perceived social anxiety in the paragraph starting on 329.

We have now extended this section to include coverage of some of the potential risks of living with (and growing up with) undiagnosed/untreated social anxiety.

-There are additional study limitations that the authors do not address in the manuscript. The most notable limitation is the use of an online survey to gather responses. The authors cannot be confident that participants are accurate in their reports of demographics and social anxiety symptoms. Additionally, the authors were only able to utilize one questionnaire of social anxiety rather a diagnostic interview and are unable to gather important information such as previous/current treatment or medication management of symptoms. Relatedly, the authors were unable to include additional measures beyond social anxiety symptoms that could have contributed to their findings, such as social media usage questionnaires.

Thank you for these suggestions, which we have now tried to address in the limitations section. In particular, we have noted the risk of inaccuracy when using self-report measures online. A diagnostic interview has been suggested for future research, but in some defence of the current approach, the SIAS has been found to reliably differentiate between clinical and non-clinical samples, so it does have utility in terms of screening for social anxiety.

-Consider including a section on the clinical implications of these findings or include these implications throughout the discussion.

The main implication of our study findings is that 18-24 year olds are a key risk group for social anxiety. It is therefore this group that require most attention and support. The paragraph prior to the study limitations section and the conclusion convey this. However, for clinical interventions (what the support entails), we are not certain what to recommend. The literature suggests cognitive behavioural approaches are generally effective, similar to adult groups. We have now cited this just prior to the limitation section when appropriate services are mentioned. However, we don’t know for sure whether social media or relationship development issues are implicated, and therefore whether they could be a focus for interventions. At the moment, we have foregrounded the at-risk group and believe that further investigation of social anxiety at this age may point to directions for clinical intervention.

Minor:

2. To improve the flow of the article, it is suggested that the authors move lines 32-34 (starting with “individuals experiencing social anxiety visibly struggle…” to after line 29 “boring or incompetent.” This allows for line 35 to continue with the authors’ discussion on impairment.

Thank you for this suggestion. We have made the change.

3. Consider adding race/ethnicity and SES to Table 1’s demographics.

We agree that this information may be useful to others who wish to consider the relationship between race/ethnicity, SES, and social anxiety. However, race/ethnicity was not explored in the original project where the dataset was created (although region was), and for SES, the best indicator appears to be estimation of household total annual income. Unfortunately, this question appears to have been poorly completed (low response rate) and for those who did respond, we would query whether individuals at the lower age range of the sample can estimate their annual household income (if still living with parents) at an accurate level.

4. On line 116, please provide which three items from the scales were not included in analyses.

These are now described immediately following the sentence about their omission.

5. The paragraph starting on line 314 continually indicates scores are “worse” or “poorly.” Consider rephrasing to clarify if authors mean score higher/lower.

These descriptors have been replaced as requested. Higher and lower are now used.

Reviewer #2: This is a well-written manuscript addressing an important topic. Strengths include the large sample, and cross-national comparisons.

The most significant limitation of the current manuscript is not carefully distinguishing between heightened social anxiety vs social anxiety disorder. Relying on cutoff scores on the SIAS greatly overestimates the prevalence of social anxiety disorder. Given that the authors report that they collected data on functioning (see page 7), I would have liked to see the authors incorporate impairment in functioning in their determination of "disorder".

Thank you for these comments, which we have tried to address in a revision of our manuscript.

In the introduction, when SAD is first mentioned, we have now stated that it involves significant impairment in functioning. Thank you for this suggestion.

We have also now addressed the issue of distinguishing between social anxiety disorder vs. subclinical/subthreshold/moderate social anxiety in the introduction, suggesting that social anxiety is becoming to be seen more commonly as a spectrum, where impairment in functioning can be significant across various life domains at levels below that of a disorder. We have noted this again briefly in the Method section (in the Analyses subsection) so the reader may understand why our results are presented in terms of SAD prevalence as well as raw SIAS scores. We hope that this goes someway towards addressing concerns about cutoff scores, and we also mention in the first paragraph of the discussion that the average SIAS scores we determined in our study exceed previous reports that include SIAS scores.

6. PLOS authors have the option to publish the peer review history of their article ([what does this mean?](https://journals.plos.org/plosone/s/editorial-and-peer-review-process#loc-peer-review-history)). If published, this will include your full peer review and any attached files.

If you choose “no”, your identity will remain anonymous but your review may still be made public.

**Do you want your identity to be public for this peer review?** For information about this choice, including consent withdrawal, please see our [Privacy Policy](https://www.plos.org/privacy-policy).

Reviewer #1: No

Reviewer #2: No
